# Ceramide kinase/ceramide 1-phosphate signaling regulates LC3B expression and autophagosome formation

**DOI:** 10.1016/j.jlr.2026.101077

**Published:** 2026-06-12

**Authors:** Hideki Funou, Natsuka Arai, Shimon Nakajima, Ryo Kadowaki, Kenta Nakamaura, Miaki Uzu, Takuya Honda, Hiroyuki Nakamura

**Affiliations:** Laboratory of Chemical Pharmacology, Graduate School of Pharmaceutical Sciences, Chiba University, Chiba, Japan

**Keywords:** sphingolipids, ceramides, cell signaling, golgi apparatus, proteomics, ceramide kinase, ceramide 1-phosphate, LC3B, autophagosome formation, Nrf2

## Abstract

Ceramide kinase (CerK) generates ceramide 1-phosphate (C1P), a bioactive sphingolipid involved in diverse cellular responses, but its role in autophagy is not fully understood. Here, we examined whether the CerK/C1P pathway regulates LC3B expression and autophagosome formation in HeLa cells. Proteomics analysis of cerebellum from *Cerk*-KO mice identified reduced levels of multiple autophagy-related proteins. In HeLa cells, genetic ablation, siRNA-mediated knockdown, and pharmacological inhibition of CerK consistently reduced LC3B-II levels. This effect was reversed by extracellular C1P and by re-expression of wild-type, but not kinase-dead, CerK, indicating that CerK-generated C1P is required for maintenance of LC3B-II. LC3B-II levels remained lower in *CERK*-KO cells in the presence of bafilomycin A1, and two-step flux analysis showed that disruption of the CerK/C1P pathway preferentially impaired the LC3B-associated autophagosome formation parameter. *MAP1LC3B* mRNA and Nrf2 protein levels were reduced in *CERK*-KO cells, and pharmacological activation of Nrf2 tended to restore *MAP1LC3B* mRNA levels and significantly increased LC3B-II protein levels. Finally, loss of the CerK/C1P pathway enhanced nutrient starvation-induced apoptotic responses and loss of viability. Together, these results identify the CerK/C1P pathway as a positive lipid signaling mechanism that maintains LC3B expression, supports LC3B-associated autophagosome formation, and promotes cell survival under nutrient-deprived conditions.

## Graphical Abstract

**Figure:**
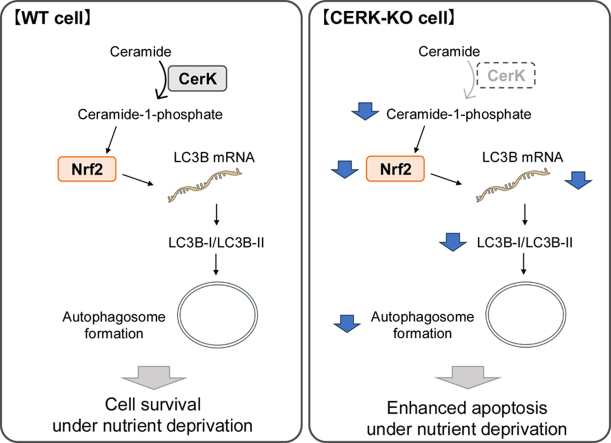

Autophagy is an evolutionarily conserved degradative process that sequesters intracellular components and delivers them to lysosomes for breakdown and recycling (1, 2). Three major forms of autophagy have been described, namely macroautophagy, microautophagy, and chaperone-mediated autophagy, all of which differ mechanistically but share lysosomes as the final degradative compartment. Among them, macroautophagy (hereafter referred to as autophagy) is characterized by the formation of double-membraned vesicles called autophagosomes, which engulf cytoplasmic cargos and subsequently fuse with lysosomes. Basal autophagic flux is essential for the turnover of proteins and organelles, whereas stress conditions such as nutrient starvation enhance autophagy to maintain cellular homeostasis and survival (1, 2). In general, autophagic flux includes initiation of phagophore formation, maturation and closure of autophagosomes, fusion with lysosomes to form autolysosomes, and degradation of intravesicular cargos. These processes are controlled by highly conserved autophagy-related proteins, including the unc-51-like autophagy activating kinase 1 (ULK1) complex, class III phosphatidylinositol 3-kinase complex I, WD repeat domain phosphoinositide-interacting protein 2 (WIPI2), and the ATG12–ATG5–ATG16L1 conjugation system, which promotes conversion of LC3B-I, the cytosolic form, to LC3B-II, the lipidated membrane-associated form (1, 2).

Sphingolipids and their metabolic enzymes have emerged as important regulators of autophagy and cell fate (3, 4). Ceramide, one of the central sphingolipids, has been implicated in both autophagy induction and stress-associated cell death, including apoptosis (3, 4, 5). In addition to ceramide itself, several sphingolipid metabolites appear to modulate autophagy-related pathways. Ceramide 1-phosphate (C1P) transfer protein (CPTP), which transfers C1P from the Golgi to the plasma membrane, has been reported to regulate autophagy, and CPTP knockdown increased LC3B expression and autophagic flux in HeLa cells (6, 7). These findings suggest that intracellular C1P metabolism is functionally linked to autophagy control. However, it remains unclear whether C1P generated by ceramide kinase (CerK), the enzyme that phosphorylates ceramide to C1P at the Golgi, directly contributes to the regulation of autophagy-related responses (8).

CerK/C1P signaling has been implicated in diverse cellular processes, including proliferation, migration, inflammation, and survival (9, 10, 11, 12). Nevertheless, its role in autophagy has not been fully defined. In particular, it remains unknown whether the CerK/C1P pathway regulates LC3B expression, autophagosome formation, and cellular adaptation to nutrient starvation. In the present study, we investigated the role of the CerK/C1P pathway in autophagy-related events using HeLa cells. We show that disruption of this pathway reduces LC3B expression, preferentially impairs autophagosome formation, and enhances starvation-induced cell death, supporting a model in which CerK-generated C1P acts as a positive lipid mediator of autophagy-related responses.

## Materials and Methods

### Cell culture, RNA interference, and plasmid transfection

HeLa cells were used as the parental cell line. *CERK*-knockout (*CERK*-KO) HeLa cells were established in our laboratory using the CRISPR-Cas9 system, as described previously (13). WT and *CERK*-KO cells were routinely cultured in DMEM (Nacalai Tesque) supplemented with 10% FBS and 1% antibiotics.

For transient knockdown of *CERK*, WT HeLa cells were transfected with control siRNA (siCtrl) or siRNA targeting *CERK* (siCERK) using Lipofectamine RNAiMAX (Thermo Fisher Scientific) according to the manufacturer’s instructions. The sequence of siCERK was 5′-G(M)CG(M)G(M)AUAUAUG(M)AAAG(M)AAAAdTdT-3′ (sense) and 5′-UUUUCUUUCAUAUAUCCGCdTdT-3′ (antisense). The sequence of siCtrl was 5′-UG(M)G(M)UUUACAUG(M)UCG(M)ACUAAdTdT-3′ (sense) and 5′-UUAGUCGACAUGUAAACCAdTdT-3′ (antisense). G(M) indicates 2′-O-methylated guanosine, and dT indicates deoxythymine. Briefly, cells cultured in 6-well plates were incubated in 1,200 μl Opti-MEM, and 200 μl Opti-MEM containing 14 pmol siRNA and 4.2 μl Lipofectamine RNAiMAX was added. To enhance knockdown efficiency, transfection was repeated every 48 h for a total of 96 h.

For rescue experiments, *CERK*-KO cells were transfected with plasmids encoding HA-tagged CerK (CerK-HA) or kinase-dead CerK-G198D-HA (14) using Lipofectamine 2000 (Thermo Fisher Scientific). After transfection for 3 h, cells were cultured in DMEM containing 10% FBS for 3 days and then selected with 0.5 mg/ml G418 for at least 10 days to establish stable cell lines. Expression of CerK-HA and CerK-G198D-HA was confirmed by Western blotting using anti-CerK and anti-HA antibodies, and CerK activity was evaluated by measuring NBD-C1P formation.

### Reagents, antibodies and plasmid

NBD-Ceramide was purchased from Thermo Fisher Scientific; BafA and DEM were from FUJIFILM Wako; NVP-231 was from Cayman; C16-C1P (Cat# 860533) was from Avanti Polar Lipids, mouse monoclonal antibody against nrf2 (Cat# sc-518033) was from Santa Cruz; GAPDH (Cat# 016-25523) was from FUJIFILM Wako; rabbit polyclonal antibody against LC3 (Cat# PM036) was from Medical & Biological Laboratories; cleaved PARP (Cat# 9541) was from Cell Signaling Technology; NRF1 (Cat# 12482-1-AP) and nrf1/NFE2L1 (Cat# 12936-1-AP) were from Proteintech; CerK was created in our laboratory; rabbit monoclonal antibody against cleaved caspase-3 (Cat# 9664) and cleaved caspase-9 (Cat# 7237) were from Cell Signaling Technology; rat monoclonal antibody against HA (Cat# 11867423001) was from Roche (Basel, Switzerland); horseradish peroxidase-conjugated anti-rabbit IgG antibody (Cat# 7074), anti-mouse IgG antibody (Cat# 7076), and anti-rat IgG antibody (Cat# 7077) were from Cell Signaling Technology. The plasmid pIRESneo3-GFP-LC3-RFP-LC3ΔG was a kind gift from Dr Noritaka Yamaguchi (Kyoto Pharmaceutical University).

### Nutrient starvation and measurements of apoptosis-related responses and cell viability

Nutrient starvation was induced by two methods. In one protocol, cells at 50% to 70% confluence were cultured in amino acid-free DMEM without FBS (amino acid/serum-free medium) for 6 h, 24 h, or 0 to 2 days. In the other protocol, cells were cultured in Hanks’ balanced salt solution (HBSS) containing 1% FBS. Control cells were maintained in DMEM containing 10% FBS.

Apoptosis-related responses were evaluated by measuring cleaved PARP, cleaved caspase-3, and cleaved caspase-9 by Western blotting. For analyses under nutrient-starved conditions, both plate-attached and floating cells were collected. Briefly, attached cells were harvested using a scraper and pooled with floating cells in the medium, followed by centrifugation at 1,000 *g* for 5 min at 4°C. Protein samples were prepared from the resulting cell pellets, and total protein concentrations were determined before SDS-PAGE. Equal amounts of protein were then subjected to electrophoresis and used for subsequent Western blot analyses.

Cell viability was assessed using a WST-8 assay kit (Cat# CK04, Dojindo), which reflects mitochondrial metabolic activity in viable cells.

### Western blotting

Cells were lysed in SDS sample buffer and sonicated using a probe-type sonicator. Proteins were separated by SDS-PAGE and transferred to PVDF membranes. After blocking, membranes were incubated with primary antibodies, followed by appropriate secondary antibodies. Immunoreactive bands were visualized as described previously (13, 15).

### qRT-PCR

Total RNA was isolated using ISOGEN II (NIPPON GENE). cDNA was prepared using 4× DN Master Mix with gDNA Remover and 5× RT Master Mix II (Toyobo, Osaka, Japan). Quantitative real-time PCR was performed using a CFX Duet RT-PCR System (Bio-Rad). The primer sequences were as follows: *CERK*, forward 5′- AGTCCACCACAACAGCAC-3′ and reverse 5′- GAGGAAGGTCTTTAAACCTG-3′; *MAP1LC3A*, forward 5′-GCTACAAGGGTGAGAAGCAGCT-3′ and reverse 5′-CTGGTTCACCAGCAGGAAGAAG-3′; *MAP1LC3B*, forward 5′-AACGGGCTGTGTGAGAAAAC-3′ and reverse 5′-AGTGAGGACTTTGGGTGTGG-3′.

### Measurements of autophagic flux by the two-step model

Autophagic flux was analyzed using the two-step model proposed by Plaza-Zabala *et al.* (16). WT and *CERK*-KO cells were cultured in DMEM containing 10% FBS and treated in the presence or absence of 10 nM BafA, an inhibitor of vacuolar H^+^-ATPase that blocks lysosomal acidification and function, for 24 h LC3B-II levels were then quantified by Western blotting, and the formation and degradation parameters were calculated according to the two-step model.

### Measurement of CerK activity in cells

CerK activity in cells was evaluated by measuring the formation of NBD-C1P from NBD-C6-ceramide. Cells were incubated in 12-well plates with 10 μM NBD-C6-ceramide for 90 min at 37°C in HBSS containing 0.1% BSA. Lipids in the wells, including both cells and medium, were extracted using the Bligh and Dyer method. Total lipid extracts were applied to Silica Gel 60 TLC plates (Merck) and separated using 1-butanol/acetic acid/water (3:1:1, v/v/v). Fluorescent lipid bands were detected using a ChemiDoc MP imaging system (Bio-Rad).

### Quantification of endogenous C1P and ceramide by LC-MS/MS

Endogenous C1P and ceramide species were quantified by LC-MS/MS as described previously (13). Briefly, lipids were extracted from WT and *CERK*-KO HeLa cells, and C1P and ceramide species were analyzed using LC-MS/MS. Lipid levels were normalized to protein content and expressed as pmol/mg protein.

### Animals

C57BL/6J *Cerk*-KO were kindly gifted by Dr Igarashi (Hokkaido University, Sapporo, Japan). Mice were bred and housed under specific pathogen-free conditions and cared for according to the animal care guidelines of Chiba University. All animal experiments were performed according to a protocol approved by the Animal Welfare Committee of Chiba University (approval no. 6-216).

### Proteomics and RNA-seq data

Proteomics and RNA-seq analyses of cerebellar tissue were outsourced to Kazusa DNA Research Institute. Cerebellar tissues were obtained from 6-8-week-old male C57BL/6J WT and *Cerk*-KO mice (n = 3 per group). Protein concentrations were determined using the BCA assay, and the samples were adjusted accordingly before further processing. Proteins were prepared using an SP3 bead-based method, digested with Trypsin/Lys-C, desalted using reverse-phase spin columns, and subjected to DIA-MS analysis using an Orbitrap Exploris 480 mass spectrometer coupled to a Vanquish Neo LC system.

The acquired DIA-MS data were analyzed using DIA-NN 2.1.0 Enterprise software. A predicted spectral library was generated using the mouse UniProtKB/Swiss-Prot database. Library-free search/library generation and deep learning-based prediction of spectra, retention times, and ion mobility were enabled. The in silico digestion parameters were as follows: enzyme, trypsin; missed cleavages, 1; N-terminal methionine excision, enabled; cysteine carbamidomethylation, enabled; peptide length range, 7 to 45 amino acids; precursor charge range, 2 to 4; precursor m/z range, 500 to 740; and fragment ion m/z range, 200 to 1800.

Protein and peptide identification and quantification were performed using DIA-NN 2.1.0 Enterprise with the predicted spectral library described above. The mass accuracy and MS1 accuracy were set to 10 ppm. Precursor and protein false discovery rates were set to ≤1%. Protein inference was enabled, and quantification was performed using the QuantUMS high-precision strategy with RT-dependent cross-run normalization. DIA-NN-derived protein abundance values were used for downstream analyses.

For heatmap visualization, z-scores were calculated from DIA-NN-derived protein abundance values across individual samples. Volcano plots were generated from log2 fold changes and adjusted *P* values calculated from the same DIA-NN-derived proteomics dataset. Statistical comparisons between WT and Cerk-KO mice were performed using multiple t-tests followed by Benjamini–Hochberg correction for multiple comparisons. Proteins with adjusted *P* values < 0.05 were considered statistically significant. Heatmaps and volcano plots were generated using GraphPad Prism 10 (version 10.5.0).

For RNA-seq data visualization, raw count data were normalized using the reads-per-million (RPM) method, followed by z-score calculation. The heatmap shown in Fig. S1B was generated using GraphPad Prism 10 (version 10.5.0).

### GFP-RFP-LC3 reporter assay

WT and *CERK*-KO HeLa cells were transfected with the GFP-RFP-LC3 reporter plasmid and selected with 0.5 mg/ml G418 to establish stable cell lines. Cells were then cultured in amino acid-free DMEM without FBS for the indicated periods. Representative fluorescence images were obtained by fluorescence microscopy, and GFP and RFP signal intensities were quantified. The GFP/RFP ratio was calculated for each condition.

### Statistical analyses

All data are presented as mean ± SEM unless otherwise indicated. For cell-based experiments, each experiment was repeated at least three times independently using separately cultured cells, and quantitative data were obtained from independent biological replicates rather than technical replicates unless otherwise stated. Representative Western blot images are shown from experiments that yielded consistent results across independent repeats. Comparisons between two groups were performed using an unpaired two-tailed Student’s *t* test. Comparisons among more than two groups were performed using one-way ANOVA followed by Bonferroni’s or Tukey’s multiple-comparison test, as appropriate. A value of *P* < 0.05 was considered statistically significant.

## Results

### Loss of the CerK/C1P pathway alters autophagy-related proteins in the cerebellum of *Cerk*-KO mice

**Fig. 1:**
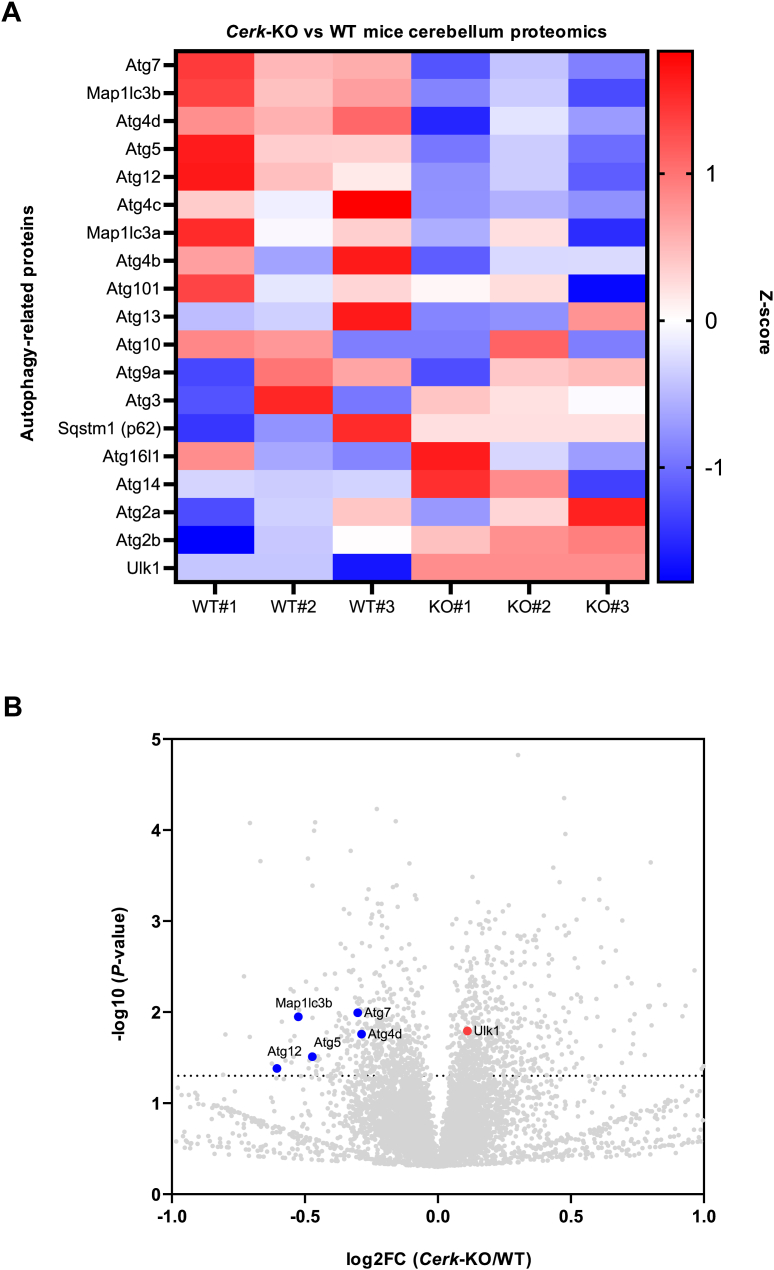
Loss of the CerK/C1P pathway alters autophagy-related proteins in the cerebellum of *Cerk*-KO mice. A: Heatmap showing z-scores calculated from DIA-NN-derived values of autophagy-related proteins in the cerebellum of WT and *Cerk*-KO mice. Three male mice (6–8 weeks old) were analyzed in each group. Blue and red indicate relative downregulation and upregulation, respectively. B: Volcano plot generated from the same DIA-NN-derived proteomics dataset. The x-axis indicates log2 fold change values for *Cerk*-KO versus WT mice, and the y-axis indicates -log10 adjusted *P* values. Proteins with adjusted *P* values < 0.05 were considered statistically significant. Detailed transcriptome data are shown in Fig. S1B.

To obtain an initial overview of molecular changes associated with loss of the CerK/C1P pathway, we analyzed proteomics data from the cerebellum of WT and *Cerk*-KO mice, with a particular focus on autophagy-related proteins. Heatmap visualization of DIA-NN-derived protein abundance values showed that several autophagy-related proteins were decreased in *Cerk*-KO mice compared with WT mice (Fig. 1A). In the same proteomics dataset, LC3B, ATG4D, ATG5, ATG7, and ATG12 showed lower abundance values in *Cerk*-KO mice, whereas ULK1 showed higher abundance (Fig. 1B). These proteins met the statistical significance criterion of adjusted *P* < 0.05 after Benjamini–Hochberg correction. In contrast, transcriptome analysis did not show similarly clear and consistent changes in the corresponding mRNAs (Fig. S1B), suggesting that loss of the CerK/C1P pathway affects autophagy-related pathways, at least in part, at the protein level. Because LC3B is a widely used marker closely associated with autophagosome formation and autophagic activity (1, 2), we focused on LC3B in the following cellular experiments using HeLa cells as a tractable cellular model.

### Loss of CerK reduces endogenous C1P and LC3B-II levels in HeLa cells

**Fig. 2:**
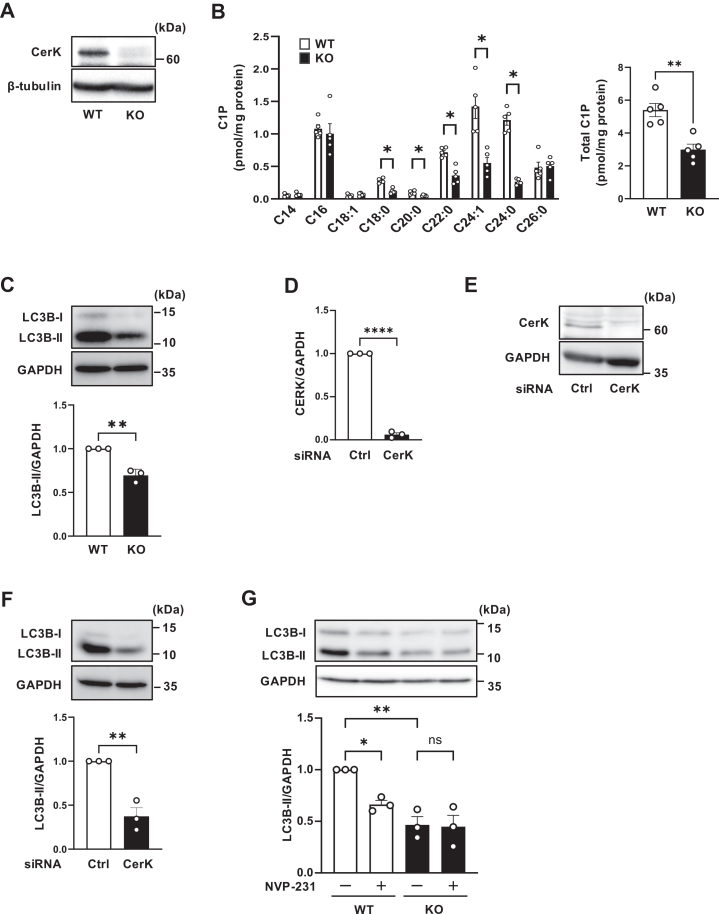
Loss of CerK reduces endogenous C1P and LC3B-II levels in HeLa cells. A: CerK protein expression in WT and *CERK*-KO HeLa cells was examined by Western blotting. B: Endogenous C1P species and total C1P levels in WT and *CERK*-KO HeLa cells were quantified by LC-MS/MS. C: Levels of LC3B-I and LC3B-II were examined in WT and *CERK*-KO HeLa cells by Western blotting. D–F: WT HeLa cells were transfected with control siRNA or siRNA against *CERK* for 96 h. Knockdown of *CERK* was confirmed by qRT-PCR (D) and Western blotting (E), and LC3B-I/II levels were examined (F). G: WT and *CERK*-KO HeLa cells were treated with 200 nM NVP-231 for 96 h, and LC3B-I/II levels were analyzed by Western blotting. NVP-231 decreased LC3B-II levels in WT cells, but not in *CERK*-KO cells. In (C, F, and G), representative immunoblots and quantitative data of LC3B-II are shown in the upper and lower panels, respectively. Data are expressed as fold change relative to the corresponding control. Data represent mean ± SEM from three independent experiments.

To confirm the cellular model used in this study, we first validated *CERK* deficiency in *CERK*-KO HeLa cells. The *CERK*-KO clone used here was the same clone previously generated by CRISPR-Cas9 and validated by genomic sequencing, which confirmed frameshift mutations introducing premature stop codons in all *CERK* alleles (13). In the present study, *CERK* deficiency was further confirmed by Western blotting (Fig. 2A). We also measured endogenous C1P levels by LC-MS/MS and confirmed that total C1P levels were reduced in *CERK*-KO cells compared with WT cells, although C1P was not completely depleted (Fig. 2B). LC-MS/MS analysis of endogenous ceramide levels showed that total ceramide levels were not significantly different between WT and *CERK*-KO cells (Fig. S2).

To examine whether the CerK/C1P pathway regulates LC3B at the cellular level, we first compared LC3B protein levels between WT and *CERK*-KO HeLa cells. Western blot analysis showed that the level of LC3B-II was significantly lower in *CERK*-KO cells than in WT cells (Fig. 2C). We next examined whether transient downregulation of *CERK* similarly affects LC3B-II levels. Knockdown of *CERK* by siRNA was confirmed by qRT-PCR (Fig. 2D) and Western blotting (Fig. 2E). Under these conditions, the level of LC3B-II was significantly reduced in *CERK*-knockdown cells compared with control cells (Fig. 2F). To further evaluate whether CerK activity is involved in maintaining LC3B-II levels, cells were treated with NVP-231, a selective inhibitor of CerK. Treatment with NVP-231 significantly decreased LC3B-II levels in WT cells, whereas no additional decrease was observed in *CERK*-KO cells (Fig. 2G). These results show that genetic ablation, siRNA-mediated knockdown, and pharmacological inhibition of CerK reduce LC3B-II levels in HeLa cells and support the idea that *CERK*-dependent C1P production contributes to LC3B-II maintenance.

### CerK activity supports LC3B-II levels when lysosomal degradation is inhibited

**Fig. 3:**
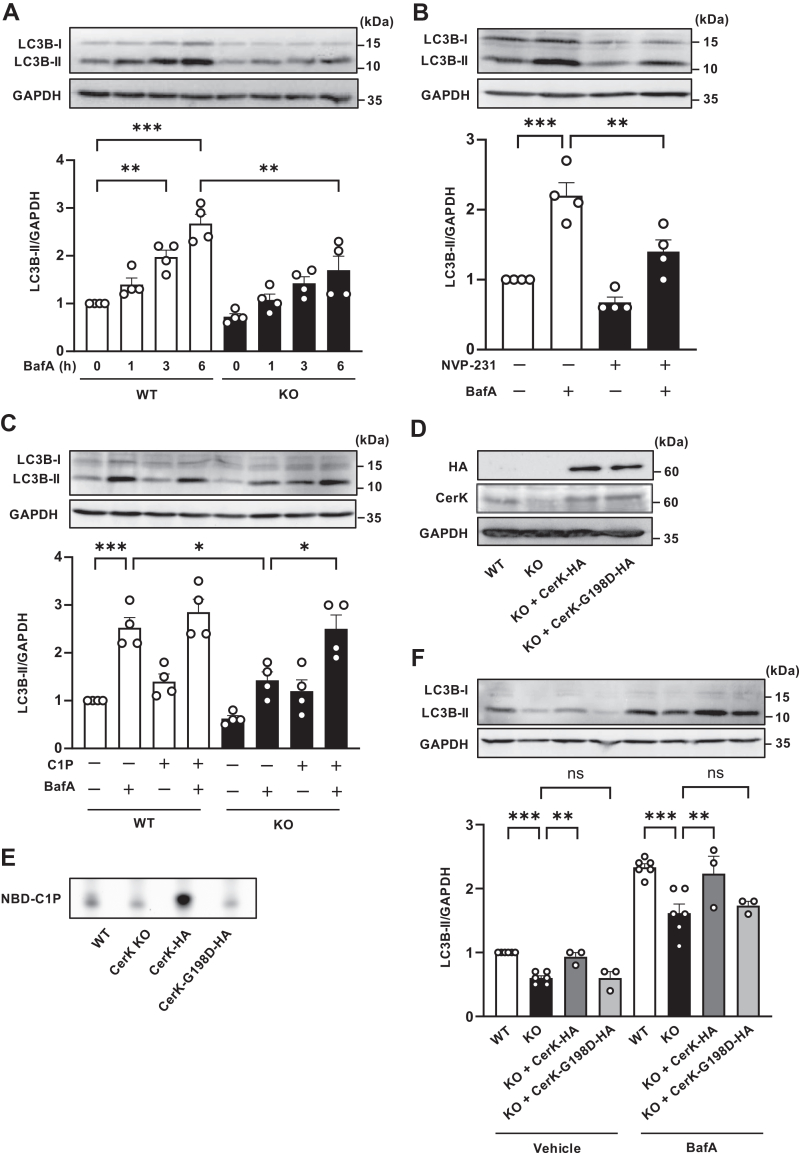
CerK activity supports LC3B-II levels when lysosomal degradation is inhibited. A: WT and *CERK*-KO HeLa cells were treated with 10 nM BafA for the indicated periods, and LC3B-I/II levels were examined by Western blotting. B: WT HeLa cells were treated with 200 nM NVP-231 for 48 h and then further incubated for 6 h in the presence or absence of 10 nM BafA. LC3B-I/II levels were analyzed by Western blotting. C: WT and *CERK*-KO HeLa cells were treated with 1 μM C1P for 6 h in the presence or absence of 10 nM BafA, and LC3B-I/II levels were examined by Western blotting. D–F: *CERK*-KO HeLa cells stably expressing WT CerK-HA or kinase-dead CerK-G198D-HA were established. Expression of CerK-HA and CerK-G198D-HA was confirmed by Western blotting using anti-HA and anti-CerK antibodies (D). CerK activity was evaluated by measuring NBD-C1P formation from NBD-ceramide (E). LC3B-I/II levels were then examined in WT cells, *CERK*-KO cells, and *CERK*-KO cells expressing WT CerK-HA or CerK-G198D-HA, in the absence or presence of BafA (F). In (A–C) and F, representative immunoblots and quantitative data of LC3B-II are shown in the upper and lower panels, respectively. Data are expressed as fold change relative to the corresponding control. Data represent mean ± SEM from three independent experiments.

Because LC3B-II itself is degraded through the autophagy-lysosome pathway (1, 2), we next examined whether the reduction of LC3B-II caused by loss of CerK could be explained simply by enhanced lysosomal degradation. To address this point, cells were treated with bafilomycin A1 (BafA), an inhibitor of vacuolar H^+^-ATPase that blocks lysosomal acidification and degradation (2). In WT cells, BafA increased LC3B-II levels in a time-dependent manner up to 6 h. However, even in the presence of BafA, LC3B-II levels remained markedly lower in *CERK*-KO cells than in WT cells (Fig. 3A), suggesting that the decrease in LC3B-II caused by *CERK* deficiency cannot be explained solely by enhanced lysosomal degradation. A similar increase in LC3B-II accumulation by BafA was also observed under 24 h treatment conditions in a concentration-dependent manner (Fig. S3). To further assess the involvement of CerK activity, WT cells were treated with NVP-231 in the presence or absence of BafA. NVP-231 decreased LC3B-II levels under both conditions (Fig. 3B).

We also examined the effect of extracellular C1P. C1P treatment tended to increase LC3B-II levels under several conditions, but a statistically significant increase was observed only in *CERK*-KO cells in the presence of BafA (Fig. 3C). Thus, although the effect of extracellular C1P was limited, these data are consistent with the possibility that C1P can contribute to LC3B-II accumulation when lysosomal degradation is blocked.

Finally, we next examined whether the enzymatic activity of CerK is required for the maintenance of LC3B-II levels. For this purpose, we established *CERK*-KO HeLa cells stably expressing WT CerK-HA or the kinase-dead CerK-G198D-HA. Expression of both proteins was confirmed by Western blotting using anti-HA and anti-CerK antibodies (Fig. 3D). Their enzymatic activities were evaluated by measuring NBD-C1P formation from NBD-ceramide. Expression of WT CerK-HA, but not CerK-G198D-HA, restored NBD-C1P formation in *CERK*-KO cells (Fig. 3E), confirming that the mutant lacked CerK activity under our experimental conditions. Consistent with this result, expression of WT CerK-HA restored LC3B-II levels in *CERK*-KO cells to levels comparable to those in WT cells, whereas expression of CerK-G198D-HA failed to do so (Fig. 3F). This rescue was also observed in the presence of BafA. These results suggest that CerK enzymatic activity, rather than CerK protein expression alone, is important for supporting LC3B-II levels. Nevertheless, because these rescue experiments involve ectopic CerK expression, we have interpreted these findings cautiously.

### Loss of the CerK/C1P pathway preferentially impairs autophagosome formation

**Fig. 4:**
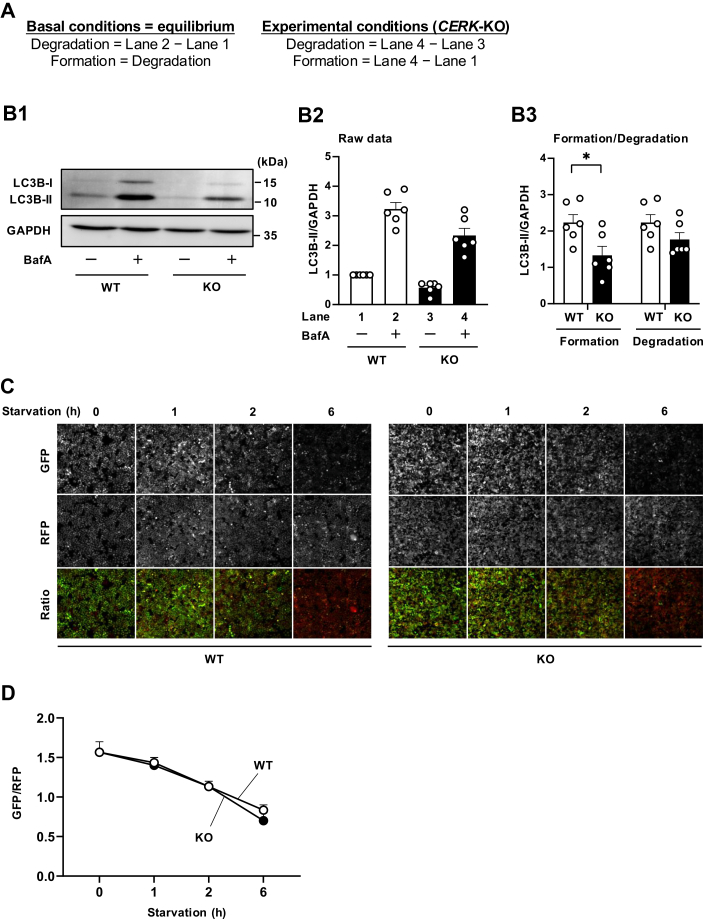
Loss of the CerK/C1P pathway preferentially impairs autophagosome formation. A: Schematic illustration of the two-step model used to estimate autophagosome formation and degradation from LC3B-II levels measured in the presence and absence of BafA. B: Autophagic flux was analyzed using the two-step model. Representative immunoblots of LC3B-I/II are shown in (B1), raw quantitative data of LC3B-II are shown in (B2), and the calculated formation and degradation parameters are shown in (B3). C: Representative fluorescence images of WT and *CERK*-KO HeLa cells expressing the GFP-RFP-LC3 reporter under nutrient starvation. D: Quantification of the GFP/RFP ratio in WT and *CERK*-KO HeLa cells under nutrient starvation. Data represent mean ± SEM from three independent experiments.

To further determine which step of autophagy is affected by disruption of the CerK/C1P pathway, we analyzed autophagic flux using a two-step model based on LC3B-II measurements in the presence and absence of BafA (16). The calculation method used to separately estimate autophagosome formation and degradation is shown in Fig. 4A. Under basal conditions, representative immunoblots of LC3B and the corresponding raw quantitative data are shown in Fig. 4B1, B2, respectively. Analysis using the two-step model showed that *CERK*-KO significantly reduced the autophagosome formation parameter, whereas the degradation parameter was only slightly decreased and did not reach statistical significance (Fig. 4B3). We next examined autophagic responses under nutrient-starved conditions using the GFP-RFP-LC3 reporter assay. Representative fluorescence images are shown in Fig. 4C, and quantitative analysis of the GFP/RFP ratio is shown in Fig. 4D. Nutrient starvation decreased the GFP/RFP ratio in WT cells, but this response was not significantly altered in *CERK*-KO cells. These results suggest that loss of the CerK/C1P pathway reduces the LC3B-based formation parameter, whereas its impact on the reporter-based autophagic response is limited under the conditions tested.

### The CerK/C1P pathway maintains *MAP1LC3B* expression, possibly through Nrf2

**Fig. 5:**
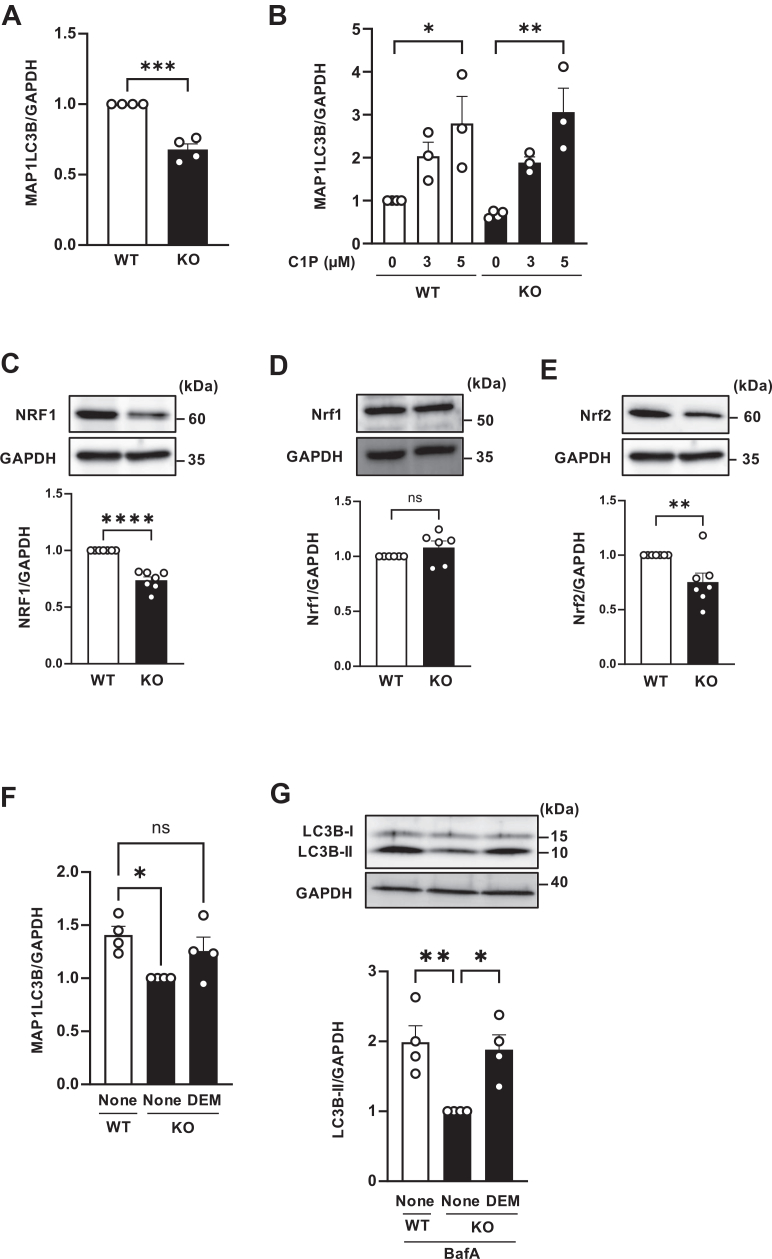
The CerK/C1P pathway maintains *MAP1LC3B* expression, possibly through Nrf2. A: *MAP1LC3B* mRNA levels were examined in WT and *CERK*-KO HeLa cells by qRT-PCR. B: WT and *CERK*-KO HeLa cells were treated with the indicated concentrations of C1P for 6 h, and *MAP1LC3B* mRNA levels were analyzed by qRT-PCR. C–E: Protein levels of NRF1 (C), Nrf1 (D), and Nrf2 (E) were examined in WT and *CERK*-KO HeLa cells by Western blotting. F, G: *CERK*-KO HeLa cells were treated with 100 μM diethyl maleate (DEM) for 24 h, and *MAP1LC3B* mRNA (F), and LC3-I/II protein levels (G) were examined. In (G), BafA was added twice (5 nM each at 0 and 12 h; final concentration, 10 nM) to reduce lysosomal degradation of LC3B-II. In (C–E and G), representative immunoblots and quantitative data are shown in the upper and lower panels, respectively. In (A, B, and F), quantitative data are expressed as fold change relative to the corresponding control. Data represent mean ± SEM from three independent experiments.

We next examined whether the reduction of LC3B-II in *CERK*-KO cells is associated with decreased *MAP1LC3B*, the gene encoding LC3B. qRT-PCR analysis showed that *MAP1LC3B* mRNA levels were significantly lower in *CERK*-KO HeLa cells than in WT cells (Fig. 5A). In addition, extracellular application of C1P increased *MAP1LC3B* mRNA levels in both WT and *CERK*-KO cells (Fig. 5B), suggesting that the CerK/C1P pathway positively regulates *MAP1LC3B* expression at the transcriptional level. Similar to *MAP1LC3B*, *MAP1LC3A* (the gene encoding LC3A) was also reduced in *CERK*-KO cells and was restored by extracellular C1P (Fig. S4).

Because nuclear factor erythroid 2-related factor 2 (Nrf2) has been reported to regulate the expression of autophagy-related genes including LC3B (17, 18, 19, 20), we next examined protein levels of nuclear respiratory factor 1 (NRF1), nuclear factor erythroid 2-related factor 1 (Nrf1/NFE2L1), and Nrf2 in WT and *CERK*-KO cells. Western blot analysis showed that the levels of NRF1 and Nrf2, but not Nrf1, were significantly lower in *CERK*-KO cells than in WT cells (Fig. 5C–E). These results raised the possibility that reduced Nrf2 expression contributes to the downregulation of *MAP1LC3B* caused by disruption of the CERK/C1P pathway. To further test this idea, *CERK*-KO cells were treated with diethyl maleate (DEM), an activator of Nrf2. In *CERK*-KO cells, DEM treatment increased *MAP1LC3B* mRNA levels to a level that was not significantly different from that in WT cells, although the difference between untreated and DEM-treated *CERK*-KO cells did not reach statistical significance (Fig. 5F). Under the same conditions, DEM significantly increased LC3B-II protein levels in *CERK*-KO cells (Fig. 5G). Together, these results suggest that the CerK/C1P pathway maintains LC3B expression, at least in part, through Nrf2 signaling.

### Loss of the CerK/C1P pathway sensitizes HeLa cells to nutrient starvation

**Fig. 6:**
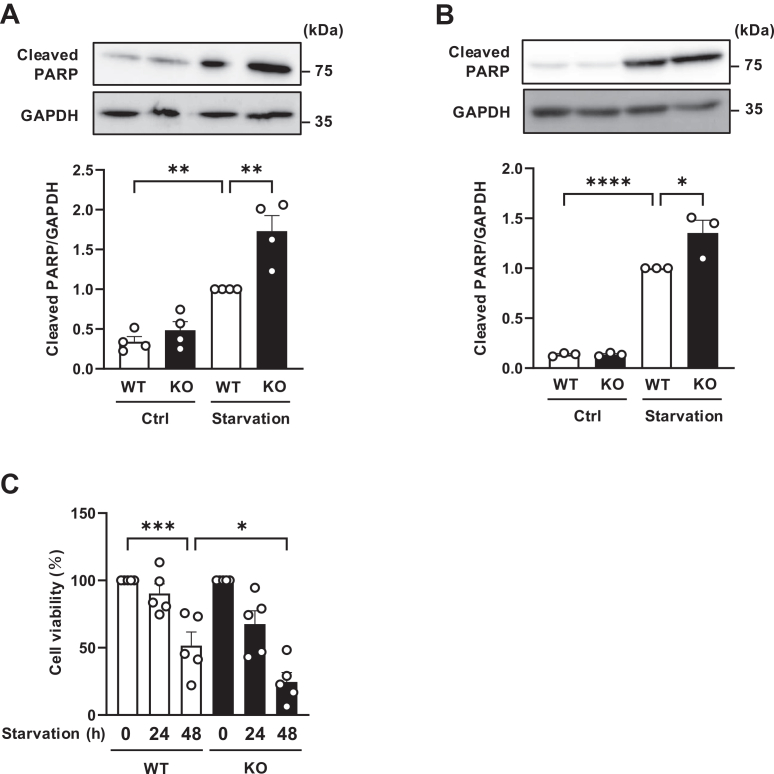
Loss of the CerK/C1P pathway sensitizes HeLa cells to nutrient starvation. A, B: WT and *CERK*-KO HeLa cells were cultured under nutrient starvation in amino acid- and serum-free medium for 6 h (A) or 24 h (B), and cleaved PARP levels were examined by Western blotting. C: WT and *CERK*-KO HeLa cells were cultured under nutrient starvation for 0 to 2 days, and cell viability was assessed using the WST-8 assay. In (A, B), representative immunoblots and quantitative data of cleaved PARP are shown in the upper and lower panels, respectively. Quantitative data are expressed as fold change relative to the value in nutrient-starved WT cells. In (C), data are expressed as fold change relative to the value in WT cells before starvation. Data represent mean ± SEM from three independent experiments.

Finally, we examined whether impairment of LC3B expression and autophagosome formation caused by disruption of the CerK/C1P pathway is associated with altered cellular responses under nutrient-deprived conditions. Under amino acid- and serum-free conditions, nutrient starvation markedly increased the levels of cleaved PARP (poly(ADP-ribose) polymerase) in WT HeLa cells at both 6 h and 24 h, and these responses were significantly enhanced in *CERK*-KO cells (Fig. 6A, B). We next assessed cell viability using the WST-8 assay. Nutrient starvation gradually reduced cell viability in WT cells, and this reduction became more pronounced in *CERK*-KO cells, especially at 48 h after starvation (Fig. 6C). These results indicate that *CERK* deficiency enhances nutrient starvation-induced loss of cell viability. A similar tendency was observed for cleaved caspase-9 under amino acid/serum starvation, although the difference did not reach statistical significance, whereas cleaved caspase-3 and cleaved PARP were significantly increased under HBSS/1% serum conditions (Fig. S5). Together, these findings suggest that the CerK/C1P pathway contributes to cell survival under nutrient-deprived conditions.

## Discussion

In the present study, we found that loss of CerK reduces LC3B expression and LC3B-associated autophagosome formation in HeLa cells. Genetic and pharmacological inhibition of CerK consistently reduced LC3B-II levels, and endogenous C1P levels were reduced in *CERK*-KO cells. LC3B-II levels were partially restored by extracellular C1P under selected conditions and by re-expression of WT, but not kinase-dead, CerK. Importantly, LC3B-II levels remained lower in *CERK*-KO cells even in the presence of BafA, suggesting that the effect of CerK deficiency is not attributable solely to enhanced lysosomal degradation. Consistent with this, analysis using the two-step model indicated that disruption of the CerK/C1P pathway preferentially impaired the LC3B-based autophagosome formation parameter. We also found that *MAP1LC3B* mRNA and Nrf2 protein levels were reduced in *CERK*-KO cells, and that pharmacological activation of Nrf2 tended to restore *MAP1LC3B* mRNA levels and significantly increased LC3B-II levels. In addition, loss of the CerK/C1P pathway enhanced starvation-induced apoptotic responses and loss of viability. These findings suggest that CerK-dependent C1P production contributes to LC3 expression and LC3B-associated autophagosome formation and may thereby support cell survival under nutrient-deprived conditions.

Sphingolipids and their metabolic enzymes have been implicated in the regulation of autophagy at multiple levels, including transcriptional regulation of autophagy-related genes and lipid-dependent modulation of autophagic membranes (3, 4). In particular, Mishra *et al.* (7) reported that knockdown of CPTP, a transfer protein for C1P, increased LC3B expression and autophagic flux in HeLa cells, supporting the idea that intracellular C1P metabolism is linked to autophagy control. In this context, our results support the idea that CerK-dependent C1P production contributes to LC3B expression and LC3B-associated autophagosome formation. Genetic and pharmacological inhibition of CerK consistently reduced LC3B-II levels, whereas extracellular C1P and re-expression of WT, but not kinase-dead, CerK restored them. Together, these findings support the view that C1P acts as a functional lipid mediator in autophagy-related regulation, rather than simply as a downstream metabolite of ceramide.

The present data further suggest that CerK-dependent C1P production supports autophagy primarily at the level of autophagosome formation rather than lysosomal degradation. LC3B-II levels remained lower in *CERK*-KO cells even in the presence of BafA, indicating that the reduction of LC3B-II cannot be explained solely by accelerated lysosomal turnover. Consistent with this interpretation, analysis using the two-step model showed that disruption of the CerK/C1P pathway preferentially reduced the autophagosome formation parameter, whereas the degradation parameter was only modestly affected. Because LC3B lipidation is required for elongation and maturation of the isolation membrane, reduced LC3B availability itself may directly limit efficient autophagosome biogenesis. Although the GFP-RFP-LC3 reporter assay did not reveal a significant difference between WT and *CERK*-KO cells under nutrient-starved conditions, this does not necessarily contradict the reduction in the LC3B-based formation parameter observed in the two-step model. The reporter assay reflects overall autophagic responses, whereas the two-step analysis in the present study specifically interrogates LC3B-II dynamics. Thus, disruption of the CerK/C1P pathway may preferentially affect LC3B-associated autophagosome formation without causing a sufficiently large global defect to be detected by the reporter system. An important limitation of the present study is that several of our conclusions are based on LC3B-related readouts. Although LC3B is a widely used marker closely associated with autophagosome formation, it does not by itself represent the entirety of the autophagy machinery. Accordingly, the present findings are best interpreted as indicating that the CerK/C1P pathway supports LC3B expression and LC3B-associated autophagosome formation, rather than establishing a universal requirement for the pathway in all forms of autophagy. Together, these findings support the idea that loss of the CerK/C1P pathway primarily compromises LC3B-associated autophagosome formation rather than causing a major defect in lysosomal degradation.

Although the rescue experiments support the involvement of CerK enzymatic activity, they should be interpreted with some caution. Extracellular C1P significantly increased LC3B-II only in *CERK*-KO cells under BafA-treated conditions, and exogenous C1P may not fully reproduce endogenous, compartmentalized C1P signaling generated by CerK. In particular, the local concentration, molecular species composition, membrane distribution, and access to signaling partners may differ between exogenous C1P and CerK-derived C1P produced at intracellular membranes. Therefore, we interpret the exogenous C1P experiments as supportive evidence that C1P can influence LC3B-related responses, rather than as direct proof that extracellular C1P fully mimics endogenous CerK-derived C1P. In addition, re-expression of WT CerK-HA restores both CerK protein and CerK activity, and therefore the contribution of CerK protein-dependent scaffolding or localization effects cannot be completely excluded. However, the failure of kinase-dead CerK-G198D-HA to restore NBD-C1P formation and LC3B-II levels supports the view that CerK enzymatic activity is important for LC3B-II maintenance.

The reduction of LC3B-II in *CERK*-KO cells also appeared to reflect decreased transcriptional support for LC3B expression. In the present study, *MAP1LC3B* mRNA levels were significantly reduced in *CERK*-KO cells and were restored by extracellular C1P, indicating that the CerK/C1P pathway positively regulates LC3B expression at the transcriptional level. A similar tendency was observed for *MAP1LC3A*, suggesting that this effect is not limited to a single LC3 isoform. Among the candidate transcriptional regulators examined, Nrf2 was decreased in *CERK*-KO cells, whereas pharmacological activation of Nrf2 tended to restore *MAP1LC3B* mRNA levels and significantly increased LC3B-II protein levels. These findings are consistent with previous reports showing that Nrf2 positively regulates autophagy-related genes, including LC3B (18, 19, 20), and support the possibility that reduced Nrf2 signaling contributes, at least in part, to the downregulation of LC3B caused by disruption of the CerK/C1P pathway. In this regard, the recent report by Dong *et al.* (21) that CerK-generated C1P stabilizes Nrf2 by interfering with the KEAP1–Nrf2 interaction provides a plausible mechanistic framework for the present findings. In contrast, ceramide itself has also been reported to promote Nrf2-dependent LC3 regulation through PKCζ/CK2-related signaling (22), suggesting that distinct branches of sphingolipid metabolism may influence autophagy through different upstream inputs to the Nrf2 axis.

In addition to the Nrf2-related mechanism discussed above, C1P may regulate LC3B-associated responses through other lipid signaling pathways. C1P has been shown to directly interact with and activate cytosolic phospholipase A2α (cPLA2α), thereby promoting arachidonic acid release and downstream eicosanoid production (23, 24). In addition, cPLA2α-initiated lipid mediator signaling has been reported to induce autophagy in macrophages and primary monocytes (25). Therefore, the C1P–cPLA2α–arachidonic acid/eicosanoid axis may also contribute to the effects observed in the present study. The current data do not distinguish whether C1P acts directly as an effector regulating LC3B expression or indirectly as an upstream initiator of cPLA2α-dependent arachidonic acid/eicosanoid signaling. Future studies using pharmacological inhibition or genetic suppression of cPLA2α will be required to determine whether cPLA2α mediates C1P-dependent regulation of MAP1LC3B expression, LC3B-II accumulation, and LC3B-associated autophagosome formation.

An important point in interpreting the present findings is whether they reflect reduced C1P levels or secondary changes in ceramide. In our previous study using the same *CERK*-KO HeLa cells, LC-MS/MS analysis showed that endogenous total C1P levels were decreased to approximately 65% of those in WT cells, whereas total ceramide levels were not significantly altered (13). In the present study, we also confirmed that endogenous C1P levels were lower in *CERK*-KO cells than in WT cells, whereas total ceramide levels were not significantly different between WT and *CERK*-KO cells. These findings support the interpretation that the present phenotype is associated primarily with reduced CerK-dependent C1P production rather than a detectable increase in bulk ceramide levels. However, the contribution of ceramide cannot be completely excluded. Because cellular ceramide levels are much higher than those of C1P and because ceramide can regulate autophagy-related pathways, small changes in specific ceramide species or compartmentalized ceramide pools may influence LC3B regulation without being evident as a significant change in total cellular ceramide. Thus, future studies using subcellular lipidomics or approaches to monitor local ceramide/C1P pools will be required to fully distinguish the contributions of reduced CerK-dependent C1P production from potential ceramide-dependent effects.

It should also be noted that *CERK* deficiency does not completely eliminate cellular C1P. Consistent with our previous study (13), residual C1P was still detected in *CERK*-KO cells in the present analysis. We previously identified DGKζ as a CerK-independent C1P-producing enzyme and showed that DGKζ contributes to selected endogenous C1P species. Thus, DGKζ may partially compensate for loss of CerK-dependent C1P production. However, DGKζ is unlikely to account for all residual C1P production, because some C1P species were not reduced by either *CERK*-KO or *DGKζ*-KO, suggesting the existence of additional CerK-independent C1P-producing pathways. Despite this residual C1P pool, CerK knockout, CerK knockdown, and pharmacological CerK inhibition all reduced LC3B-II levels, and re-expression of WT CerK, but not kinase-dead CerK, restored LC3B-II. These findings suggest that CerK-derived C1P has a non-redundant role in LC3B regulation. One possible explanation is that CerK- and DGKζ-derived C1P pools differ in their subcellular localization, molecular species composition, or local signaling partners. Future studies using CerK/DGKζ double-deficient cells or DGKζ inhibition will be required to define the contribution of CerK-independent C1P production to LC3B-associated autophagosome formation.

The present findings also distinguish CerK deficiency from previously reported effects of CPTP knockdown on autophagy regulation. CPTP knockdown increased LC3B expression and autophagic flux in HeLa cells (7), whereas loss of *CERK* in the present study decreased LC3B expression and preferentially impaired the LC3B-associated autophagosome formation parameter. These differences suggest that C1P does not act as a simple on/off signal for autophagy, but rather that its effects depend on how cellular C1P metabolism is perturbed, potentially including changes in its subcellular distribution as well as its total level. This interpretation is also consistent with recent evidence that C1P contributes to regulation of Golgi structure in a CerK-dependent manner (26), further supporting the idea that compartment-specific C1P signaling may influence downstream autophagy-related responses. Thus, distinct perturbations of C1P metabolism may have different consequences for autophagy depending on whether they alter C1P production, trafficking, or compartmental distribution. It should also be noted that the proteomics analysis identified changes not only in LC3B but also in multiple proteins involved in the ATG8ylation-related machinery, including ATG4D, ATG5, ATG7, and ATG12. Therefore, the CerK/C1P pathway may influence a broader ATG8ylation-related network rather than LC3B alone. In the present study, however, we focused on LC3B because it showed consistent changes at both the mRNA and protein levels and could be examined mechanistically in the subsequent cellular experiments. The cerebellar proteomics analysis was used here to provide an in vivo overview of molecular changes associated with loss of the CerK/C1P pathway, whereas the HeLa cell system allowed mechanistic interrogation of LC3B regulation under experimentally controlled conditions. At the same time, the present study has several limitations. Our mechanistic analyses were performed mainly in HeLa cells, and the direct molecular link between CerK-generated C1P and Nrf2-dependent transcriptional control of *MAP1LC3B* remains to be established. In addition, the cerebellar proteomics data and the HeLa cell data were not fully concordant for all autophagy-related molecules, suggesting that the impact of the CerK/C1P pathway may vary depending on cell type or tissue context. Another limitation is that we did not directly test whether exogenous C1P rescues the enhanced starvation-induced apoptotic responses or loss of viability observed in *CERK*-KO cells. Although exogenous C1P increased LC3B-II levels under some conditions, nutrient starvation-induced cell death is likely regulated by multiple pathways, and reduced LC3B-associated autophagosome formation may represent only one component of the survival defect caused by loss of CerK. Therefore, future studies examine whether exogenous C1P restores cell viability and suppresses apoptotic responses under nutrient-starved conditions will be important to determine the extent to which the survival phenotype is mediated by C1P-dependent regulation of LC3B expression and autophagosome formation. Nevertheless, our results identify CerK-dependent C1P production as a lipid signaling mechanism that supports LC3B expression, LC3B-associated autophagosome formation, and cell survival under nutrient-deprived conditions.

In summary, the present study supports a model in which CerK-dependent C1P production contributes to the maintenance of LC3B expression and LC3B-associated autophagosome formation, thereby supporting cell survival under nutrient-deprived conditions. These findings link sphingolipid metabolism to LC3B-related autophagy regulation and suggest that CerK-derived C1P has a non-redundant role in this process.

### Data availability

All data described in this manuscript are contained within the article and its Supplementary information.

## Supplemental data

This article contains supplemental data.

## Conflict of interest

The authors declare that they have no conflicts of interest with the contents of this article.
