# Supplementary material for: Ceramide kinase/ceramide 1-phosphate signaling regulates LC3B expression and autophagosome formation
Source: J Lipid Res. 2026 Jun 12;67(7):101077. doi: 10.1016/j.jlr.2026.101077 (PMC13380049; doi:10.1016/j.jlr.2026.101077)
Supplement: Supplemental Data [file mmc1.pdf]

Supporting information

Supplementary Methods

Visualization of RNA-seq data. The raw count data were normalized using the reads-per-million (RPM) method, followed by z-score calculation as described above. Visualization was performed using GraphPad Prism 10 (ver. 10.5.0). The calculated RNA-seq data are shown in Fig. S1B.

Supplementary figure legends

Fig. S1. Proteome and transcriptome analyses of autophagy-related molecules in the cerebellum of *Cerk*-KO mice.

A, Overview of proteomics data for autophagy-related proteins in the cerebellum of WT and *Cerk*-KO mice. Fold change values were calculated from three individuals in each group, and adjusted *P* values were obtained using multiple *t*-tests followed by Benjamini-Hochberg correction. Significantly decreased proteins, including LC3B, ATG4D, ATG5, ATG7, and ATG12, and significantly increased ULK1 are indicated. **B**, Heatmap showing z-scores of autophagy-related transcripts in the cerebellum of WT and *Cerk*-KO mice based on RNA-seq data. Blue and red indicate relative downregulation and upregulation, respectively. In contrast to the proteomics data, transcriptome analysis did not show clear and consistent changes in the corresponding mRNAs.

Fig. S2. Endogenous ceramide levels in WT and *CERK*-KO cells.

WT and *CERK*-KO HeLa cells were analyzed by LC-MS/MS to quantify endogenous ceramide levels. Levels of individual ceramide species and total ceramide levels are shown. Total ceramide levels were not significantly different between WT and *CERK*-KO cells. Data represent mean ± SEM from independent experiments.

Fig. S3. **BafA increases LC3B-II accumulation in a concentration-dependent manner, but LC3B-II remains lower in *CERK*-KO cells.**

WT and CERK-KO HeLa cells were treated with the indicated concentrations of BafA for 24 h, and LC3B-I/II

levels were examined by Western blotting. In both cell types, BafA increased LC3B-II accumulation in a concentration-dependent manner, whereas LC3B-II levels remained lower in *CERK*-KO cells than in WT cells. Representative immunoblots and quantitative data of LC3B-II are shown in the upper and lower panels, respectively. Data are expressed as fold change relative to the corresponding control.

<u>Fig. S4.</u> The CerK/C1P pathway positively regulates *MAP1LC3A* mRNA expression in HeLa cells.

WT and *CERK*-KO HeLa cells were treated with or without 1 μM C1P for 6 h, and *MAP1LC3A* mRNA levels were examined by qRT-PCR.

<u>Fig. S5.</u> Loss of the CerK/C1P pathway enhances additional apoptotic responses under nutrient starvation.

A, WT and *CERK*-KO HeLa cells were cultured in amino acid- and serum-free medium for 24 h, and cleaved caspase-9 levels were examined by Western blotting. **B**, **C**, WT and *CERK*-KO HeLa cells were cultured in HBSS containing 1% serum for 24 h, and cleaved caspase-3 (B) and cleaved PARP (C) levels were examined by Western blotting. In A-C, representative immunoblots and quantitative data are shown in the upper and lower panels, respectively. Quantitative data are expressed as fold change relative to the value in nutrient-starved WT cells.

A

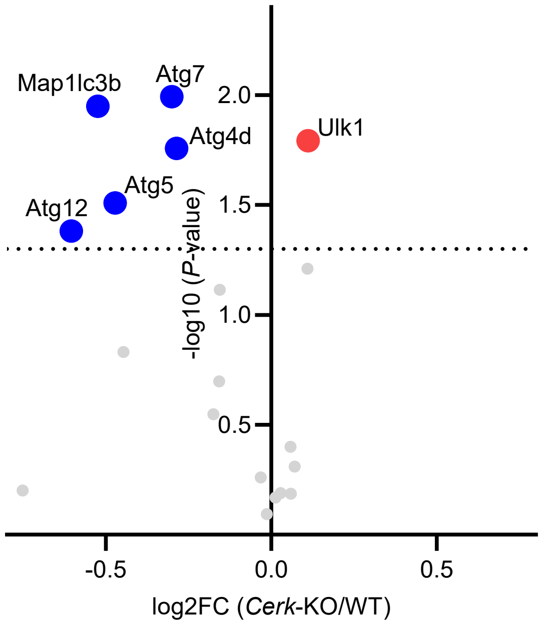

Map1lc3b
Atg7
Atg4d
Ulk1
Atg5
Atg12
2.0
1.5
1.0
0.5
-log10 (*P*-value)
-0.5
0.0
0.5
log2FC (*Cerk*-KO/WT)

B

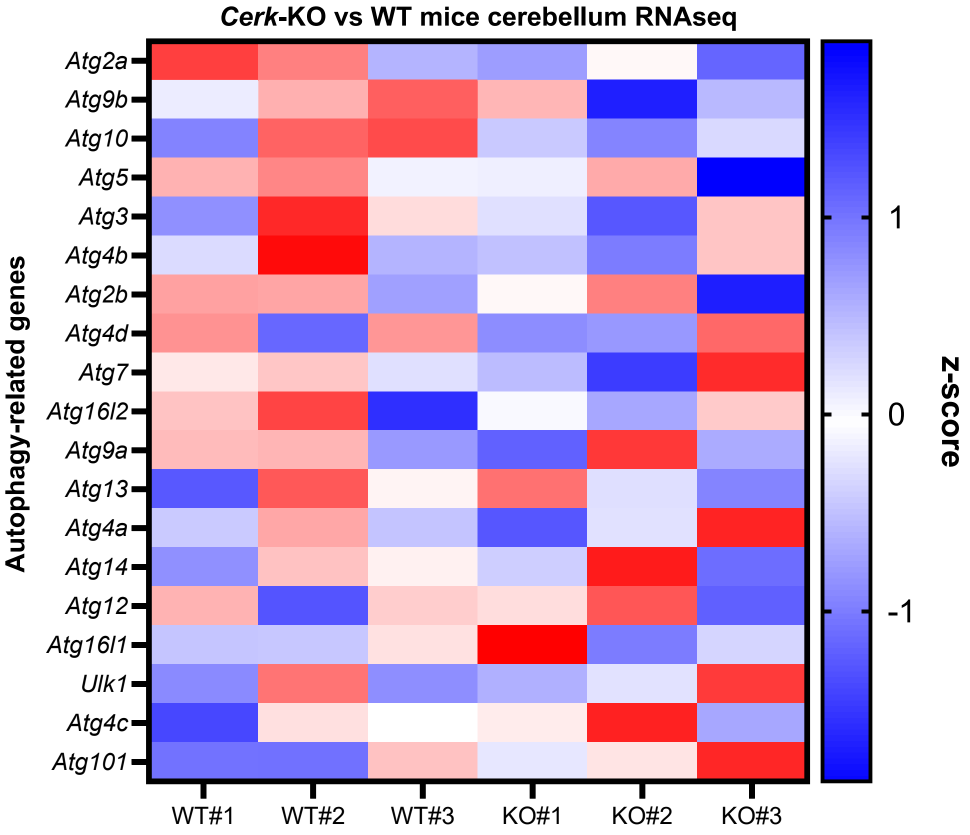

Cerk-KO vs WT mice cerebellum RNAseq
Autophagy-related genes
Atg2a
Atg9b
Atg10
Atg5
Atg3
Atg4b
Atg2b
Atg4d
Atg7
Atg16l2
Atg9a
Atg13
Atg4a
Atg14
Atg12
Atg16l1
Ulk1
Atg4c
Atg101
WT#1
WT#2
WT#3
KO#1
KO#2
KO#3
1
0
-1
z-score

Fig. S1

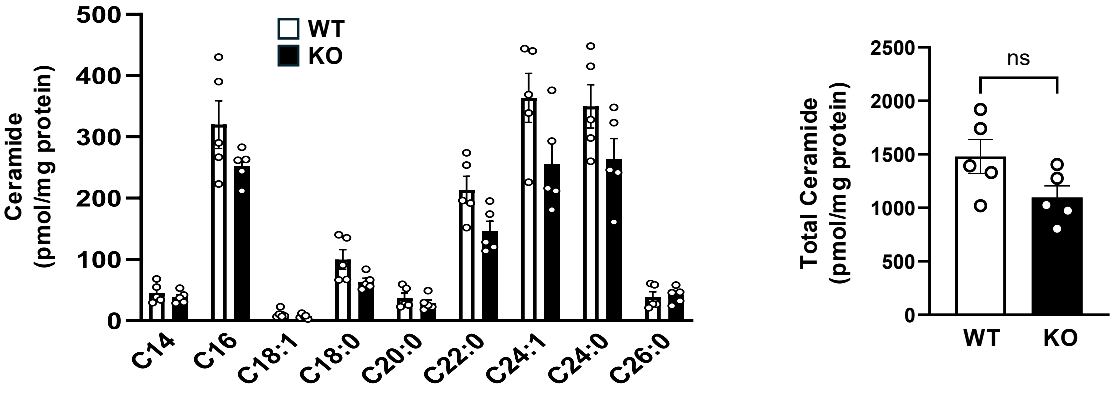
500
400
300
200
100
0
Ceramide
(pmol/mg protein)
WT
KO
C14
C16
C18:1
C18:0
C20:0
C22:0
C24:1
C24:0
C26:0
2500
2000
1500
1000
500
0
Total Ceramide
(pmol/mg protein)
ns
WT
KO

Fig. S2

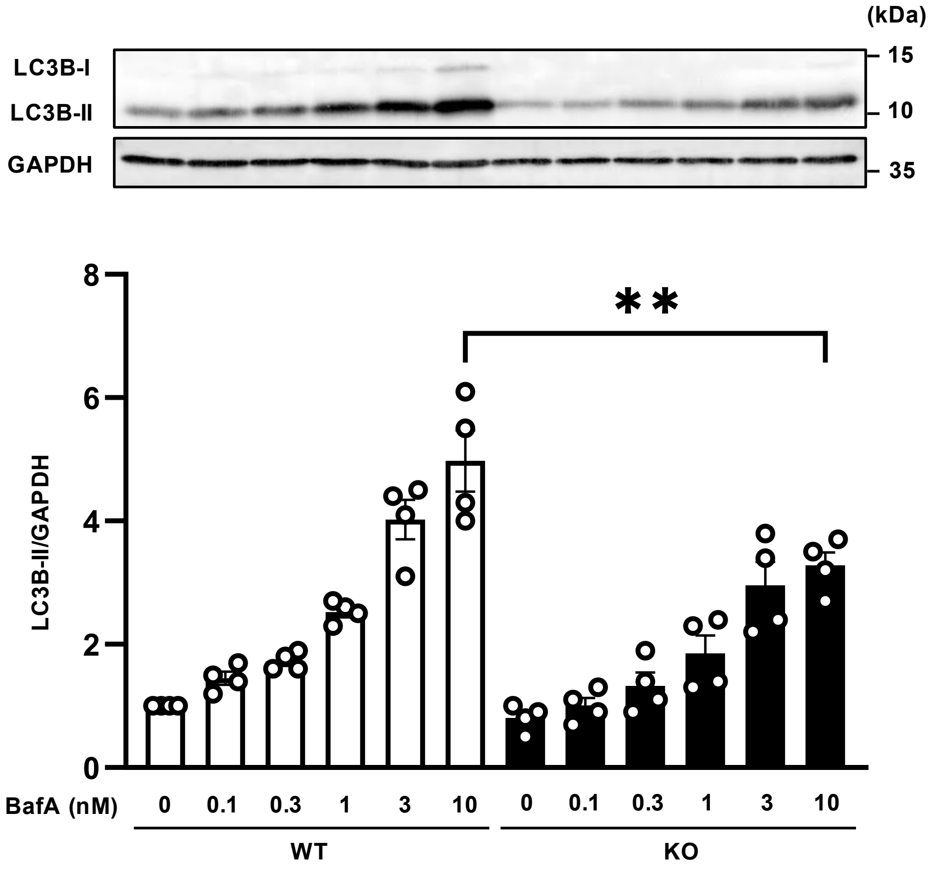

(kDa)
LC3B-I
LC3B-II
GAPDH
15
10
35
8
6
4
2
0
LC3B-II/GAPDH
**
BafA (nM)
0
0.1
0.3
1
3
10
0
0.1
0.3
1
3
10
WT
KO

Fig. S3

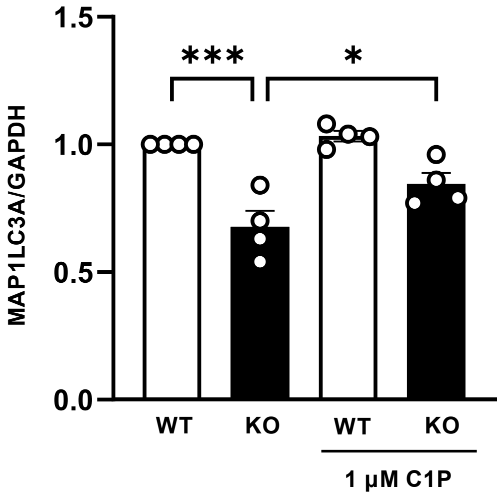

Fig. S4

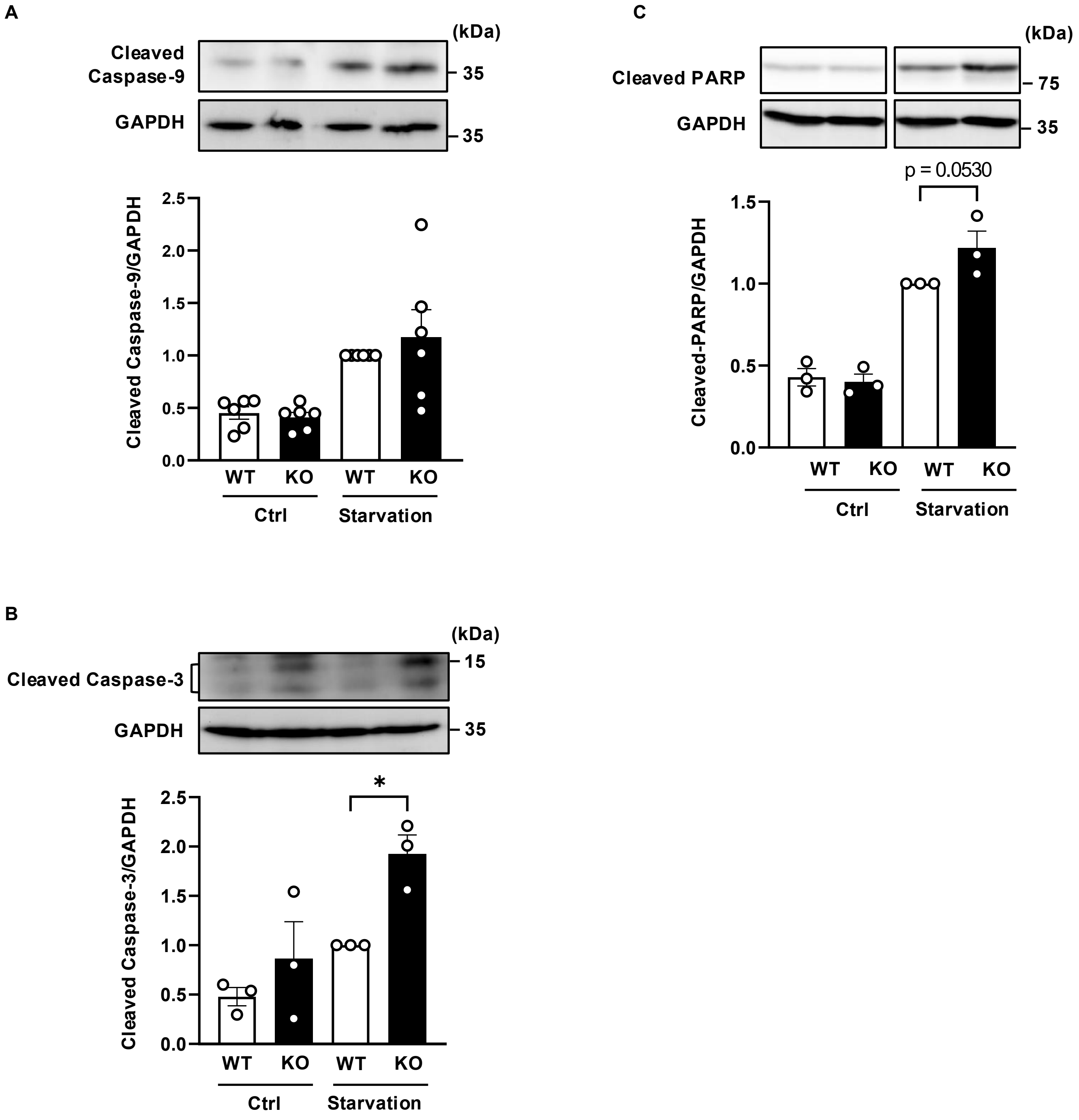
A
(kDa)
Cleaved Caspase-9
35
GAPDH
35
Cleaved Caspase-9/GAPDH
2.5
2.0
1.5
1.0
0.5
0.0
WT
KO
WT
KO
Ctrl
Starvation
B
(kDa)
Cleaved Caspase-3
15
GAPDH
35
*
Cleaved Caspase-3/GAPDH
2.5
2.0
1.5
1.0
0.5
0.0
WT
KO
WT
KO
Ctrl
Starvation
C
(kDa)
Cleaved PARP
75
GAPDH
35
p = 0.0530
Cleaved-PARP/GAPDH
1.5
1.0
0.5
0.0
WT
KO
WT
KO
Ctrl
Starvation

Fig. S5
